# Transcriptional profiling specifies the pathogen-specific human host response to infectious keratitis

**DOI:** 10.3389/fcimb.2023.1285676

**Published:** 2024-01-11

**Authors:** Thabo Lapp, Paola Kammrath Betancor, Günther Schlunck, Claudia Auw-Hädrich, Philip Maier, Clemens Lange, Thomas Reinhard, Julian Wolf

**Affiliations:** ^1^ Eye Center, Medical Center, Faculty of Medicine, University of Freiburg, Freiburg im Breisgau, Germany; ^2^ Ophtha-Lab, Department of Ophthalmology, St. Franziskus Hospital, Münster, Germany; ^3^ Omics Laboratory, Stanford University, Palo Alto, CA, United States; ^4^ Department of Ophthalmology, Byers Eye Institute, Stanford University, Palo Alto, CA, United States

**Keywords:** RNA sequencing, infectious keratitis, keratoplasty, human corneal tissue, host response, FFPE (formalin fixed paraffin embedded), viral keratitis, bacterial keratitis

## Abstract

**Purpose:**

Corneal infections are a leading cause of visual impairment and blindness worldwide. Here we applied high-resolution transcriptomic profiling to assess the general and pathogen-specific molecular and cellular mechanisms during human corneal infection.

**Methods:**

Clinical diagnoses of herpes simplex virus (HSV) (n=5) and bacterial/fungal (n=5) keratitis were confirmed by histology. Healthy corneas (n=7) and keratoconus (n=4) samples served as controls. Formalin-fixed, paraffin-embedded (FFPE) human corneal specimens were analyzed using the 3’ RNA sequencing method Massive Analysis of cDNA Ends (MACE RNA-seq). The cellular host response was investigated using comprehensive bioinformatic deconvolution (xCell and CYBERSORTx) analyses and by integration with published single cell RNA-seq data of the human cornea.

**Results:**

Our analysis identified 216 and 561 genes, that were specifically overexpressed in viral or bacterial/fungal keratitis, respectively, and allowed to distinguish the two etiologies. The virus-specific host response was driven by adaptive immunity and associated molecular signaling pathways, whereas the bacterial/fungal-specific host response mainly involved innate immunity signaling pathways and cell types. We identified several genes and pathways involved in the host response to infectious keratitis, including CXCL9, CXCR3, and MMP9 for viral, and S100A8/A9, MMP9, and the IL17 pathway for bacterial/fungal keratitis.

**Conclusions:**

High-resolution molecular profiling provides new insights into the human corneal host response to viral and bacterial/fungal infection. Pathogen-specific molecular profiles may provide the foundation for novel diagnostic biomarker and therapeutic approaches that target inflammation-induced damage to corneal host cells with the goal to improve the outcome of infectious keratitis.

## Introduction

Corneal infections are a leading cause of visual impairment worldwide, with an increasing trend that is driven by the widespread use of contact lenses (Flaxman et al., 2017; Tran et al., 2020; GBD 2019 Blindness and Vision Impairment Collaborators and Vision Loss Expert Group of the Global Burden of Disease Study, 2021; Ung and Chodosh, 2021). In most cases, bacteria and viruses are causative, whereas fungi and protozoa are found less frequently, although the spectrum of pathogens varies considerably depending on climate and geographical location (Porth et al., 2019). If appropriate therapy is not provided aggressively, infectious keratitis can lead to sight-threatening scarring or corneal perforation, which in many cases results in the loss of the eye. To develop an appropriate treatment plan for each patient, a precise diagnostic assessment is required. In current clinical routine, the diagnosis is usually based on clinical findings, leading to empirical treatment, at least initially. Emergency corneal transplantation is the only way to obtain large amounts of tissue from active corneal infection for histological and microbiological examination. Other complementary diagnostic techniques include corneal epithelial scraping, polymerase chain reaction (PCR) and in vivo confocal microscopy, albeit the latter with low sensitivity (Hudson et al., 2022; Zemba et al., 2022).

Despite growing burden of the disease, therapeutic options for infectious keratitis remain sparse. Depending on the underlying pathogen, topical antibiotics, antivirals or antifungals, along with corticosteroids are the only evidence-based treatments that have shown efficacy (Ung and Chodosh, 2021). However, increasing rates of resistance significantly affect outcomes (Austin et al., 2017). If the corneal infection cannot be controlled with medication, emergency corneal transplantation (keratoplasty à chaud) is required to save sight, in which the infected corneal tissue is surgically removed and a donor corneal graft is sutured to the recipient eye (Mathews et al., 2018). Even if the acute infection can be managed, the local inflammatory response may not only eradicate the causative pathogen, but cause collateral damage to corneal stroma cells leading to corneal scarring, a common problem that opacifies the cornea and causes significant visual impairment (Ung and Chodosh, 2021). This indicates that the right balance between the pro-inflammatory response to eliminate the pathogen and anti-inflammatory control to prevent damage to host cells is crucial and that therapeutic modulation of this balance may represent a promising approach to improve the outcome of infectious keratitis.

The general and pathogen-specific molecular and cellular mechanisms in the human cornea during infection are currently poorly understood and the poor disease outcome urgently requires novel therapeutic approaches. Previous studies demonstrated the value of RNA sequencing (RNA-seq) to examine disease mechanisms at high resolution and in an unbiased approach (Wolf et al., 2023). The technology enables detailed molecular characterization of human corneal tissue (Collin et al., 2021), even in formalin-fixed and paraffin-embedded (FFPE) corneal specimens, that are available from histological biobanks (Boneva et al., 2020; Wolf et al., 2022b). In particular, the cellular (especially immunological) compartments have been described (Wolf et al., 2020; Zhuang et al., 2022) and it has been shown how the cellular composition of corneal grafts, the tissue that will later be used for corneal transplantation, is altered by tissue culture in eye bank storage (Zhuang et al., 2022; Wolf et al., 2022b). Using microarray technology, another study demonstrated gene expression changes in bacterial and fungal keratitis (Chidambaram et al., 2017). However, the more recent availability of RNA sequencing techniques provides several advantages over microarray technology, including a more accurate and unbiased assessment of the gene expression profile. In keratoconus, a disease that affects the corneal shape, other studies have found a correlation between mechanical shear forces, expression of several protease genes, abnormal corneal epithelial differentiation and inhibition of anti-inflammatory cell signaling pathways (Dou et al., 2022). Some of these findings can now be accessed in online atlases (Wolf et al., 2022a) and represent the foundation to examine corneal infectious disease.

Here we applied high-resolution transcriptomic profiling using RNA-seq to assess the general and pathogen-specific molecular and cellular mechanisms during human corneal infection. Our results demonstrate pathogen-specific patterns of cell types and transcription, which allow to distinguish bacterial/fungal from viral keratitis and provide new insights into the pathophysiology and the host response to infectious corneal disease. These findings provide a foundation for the assessment of novel diagnostic biomarkers and therapeutic approaches that target inflammation-induced damage to corneal host cells and improve the outcome of infectious keratitis.

## Materials and methods

### Ethics

Written informed consent was obtained from all participating patients. Ethical approval was granted from the Research Ethics Committee of the University of Freiburg, Germany (reference number: 434/20). This study complies with the tenets of the Declaration of Helsinki.

### Human corneal tissue procurement

A total of 14 corneal specimens from 14 patients who underwent corneal transplantation at the Eye Center of the University of Freiburg, Germany, were included in this study (**Table 1**). The indication for surgery was made by an experienced cornea consultant. 7 healthy corneas from 7 patients who underwent enucleation due to choroidal melanoma at our institution served as controls. After surgery, corneal tissue was immediately fixed in formalin and processed for paraffin embedding followed by routine histopathological examination. The clinical diagnosis was confirmed histologically by at least two experienced ophthalmic pathologists (**Supplementary Figure 1**). There was no evidence of tumor-induced alterations in any of the healthy corneal specimens.

**Table 1:** Specimen Donor details.

Sample	Age	Eye	Sex	Clinical diagnosis	Histological diagnosis	Reads (Mio)
**Control S1**	52	OD	M	Choroidal melanoma without anterior segment involvement	Healthy corneal tissue	3,3
**Control S2**	61	OS	M			7,5
**Control S3**	54	OD	F			5,4
**Control S4**	73	OD	F			5,4
**Control S5**	74	OD	F			2,8
**Control S6**	58	OS	M			6,2
**Control S7**	56	OD	F			4,1
**Keratoconus S1**	26	OS	M	Keratoconus	Keratoconus	4,1
**Keratoconus S2**	54	OS	F			3,0
**Keratoconus S3**	57	OS	M			2,2
**Keratoconus S4**	45	OS	M			4,2
**Herpes S1**	61	OS	M	Herpetic keratitis	Chronic keratitis compatible with HSV infection	3,8
**Herpes S2**	66	OS	M	Herpetic keratitis, corneal scar	Corneal scar compatible with HSV infection	3,8
**Herpes S3**	27	OS	F	Herpetic keratitis	Active herpetic keratitis	2,4
**Herpes S4**	50	OD	F	Herpetic keratitis, corneal ulcer	Herpetic keratitis, corneal scar	4,3
**Herpes S5**	65	OD	M	Herpetic keratitis, corneal scar	Herpetic keratitis, scaring	2,7
**Bacterial/fungal S1**	62	OS	M	Acute infectious keratitis	Acute bacterial keratitis	2,2
**Bacterial/fungal S2**	65	OS	F	Acute infectious keratitis most likely due to fungi	Fungal keratitis	0,4
**Bacterial/fungal S3**	73	OS	F	Acute corneal perforation; acute keratitis	Perforated corneal ulcer in active bacterial keratitis	7,4
**Bacterial/fungal S4**	56	OD	M	Corneal ulcer and acute infectious keratitis	Corneal ulcer in acute bacterial keratitis	3,8
**Bacterial/fungal S5**	42	OD	F	Acute infectious keratitis	Acute keratitis with corneal perforation	4,6

Reads (Mio): number of sequencing raw reads in million.

### Formalin-fixation and paraffin-embedding

Corneal tissue processing was performed as previously described (Wolf et al., 2022b). Briefly, after corneas were retrieved, the tissue was fixed in 4% formalin (phosphate-buffered, pH 7) for 12 hours at room temperature, dehydrated in alcohol, and processed for paraffin embedding.

### Tissue processing

Fifteen FFPE sections, each 4 µm thick, were taken from each corneal sample and stored in RNase-free tubes prior to RNA extraction, as previously described (Wolf et al., 2022b).

### RNA extraction

RNA extraction from corneal samples was performed by a commercial provider (GenXPro, Frankfurt am Main, Germany) as previously described (Wolf et al., 2022b). Briefly, total RNA was isolated from FFPE samples using the Quick-RNA FFPE Kit (Zymo Research, Irvine, CA, USA). Following a DNAse I digestion using the Baseline-ZERO kit (Epicentre, Madison, WI, USA), the RNA concentration was measured with the Qubit RNA HS Assay Kit on a Qubit Fluorometer (Life Technologies, Carlsbad, CA, USA). The RNA quality was determined with the RNA Pico Sensitivity Assay on a LabChip GXII Touch (PerkinElmer, Waltham, MA, USA).

### RNA sequencing

RNA sequencing was performed by GenXPro using massive analysis of cDNA ends (MACE), a 3′-RNA sequencing method, as previously described (Wolf et al., 2022c). We recently demonstrated that MACE allows sequencing of FFPE samples with high accuracy (Boneva et al., 2020). Briefly, barcoded libraries comprising unique molecule identifiers were sequenced on the NextSeq 500 (Illumina, San Diego, CA, USA) with 1 × 75 bp. PCR bias was removed using unique molecular identifiers.

### Bioinformatics

Sequencing data (fastq-files) were uploaded to the Galaxy web platform (usegalaxy.eu) (Galaxy Community, 2022), as previously described (Wolf et al., 2022c). Quality control was performed with FastQC (Galaxy Version, 0.73, http://www.bioinformatics.babraham.ac.uk/projects/fastqc/last access on 6^th^ November 2022). Reads were mapped to the human reference genome (Gencode, GRCh38.p13, all regions, release 42) with RNA STAR (Galaxy Version 2.7.8a) (Dobin et al., 2013) using the corresponding Gencode main annotation file. Reads mapped to the reference genome were counted with featureCounts (Galaxy Version 2.0.1) (Liao et al., 2014) using the aforementioned annotation files. The outputs of featureCounts were imported to RStudio (Version 1.4.1103). Gene symbols were determined based on the ENSEMBL database (release 108, download on 5 November 2022) (Cunningham et al., 2022). After principle component analysis (Love et al., 2014), normalized reads and differential gene expression were calculated using the R package DESeq2 (version 1.34.0) with default parameters (Benjamini–Hochberg adjusted *p*-values) (Love et al., 2014). Transcripts with log2 fold change (log2FC) > 2 or <−2 and adjusted *p*-value < 0.05 were considered as differentially expressed genes (DEG). To determine bacterial/fungal- and viral-specific genes, we first determined DEG in bacterial/fungal and viral keratitis each compared to control corneas. Bacterial/fungal-specific genes were defined as genes that were only significantly upregulated in bacterial/fungal but not in viral keratitis. Similarly, viral-specific genes were defined as genes that were only significantly upregulated in viral but not in bacterial/fungal keratitis. The same logic was applied for downregulated genes. To identify the best candidate biomarkers for viral and bacterial/fungal keratitis, we performed receiver operating curve (ROC) analyses for each of the 216 viral as well as the 561 bacterial/fungal genes using the pROC R package (version 1.18.5). Heatmaps were created with the R package ComplexHeatmap (version 2.10.0) (Gu et al., 2016). Other data visualization was done using the ggplot2 package (version 3.3.5). Cell-type-specific marker genes for corneal cell types were determined based on previously published single-cell RNA sequencing data of the human cornea (Collin et al., 2021). Cell-type enrichment analysis was performed using xCell (Aran et al., 2017) and CYBERSORTx (using the included LM22 immune cell signature) (Newman et al., 2019). xCell uses the transcriptomic signatures of 64 distinct immune and stroma cell types to estimate the relative contributions of these cells to a bulk RNA transcriptome. Transcripts per million were calculated as an input for the analysis based on the output of featureCounts (assigned reads and feature length), as previously described (Wagner et al., 2012). xCell and CYBERSORTx enrichment scores were compared between different groups using the Mann-Whitney U-test. Functional enrichment analysis was performed using ClueGO (Bindea et al., 2009) in Cytoscape (Shannon et al., 2003) based on the following pathway databases: GO BP, KEGG, reactome pathways, Wiki pathways.

## Results

### Corneal infection induces a pathogen-specific transcriptional profile in the human cornea

**Figure 1:**
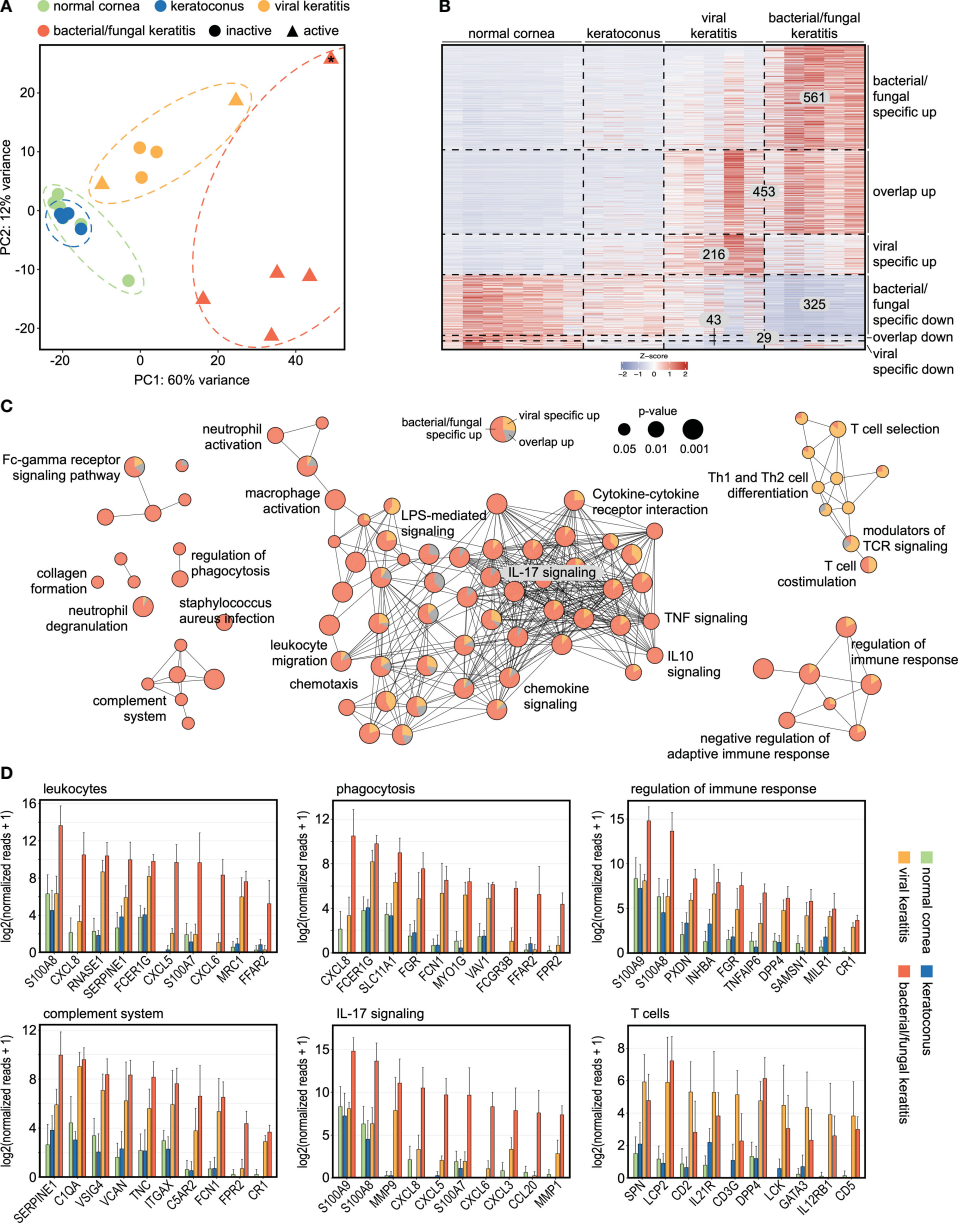
Transcriptional profiling reveals specific marker genes and associated pathways in bacterial/fungal and viral corneal infection. **(A)** The substantial effect of bacterial/fungal and viral corneal infection is illustrated by unsupervised cluster analysis using principal component analysis. Each point represents one sample. 2 of the 5 viral cases demonstrated active inflammation (triangles), whereas the remaining 3 cases were inactive (circles). The sample that is marked with an asterisk (*) was a fungal keratitis. PC: principal component. **(B)**: Heatmap visualizing 561 genes which were specifically overexpressed in bacterial/fungal keratitis, 453 genes which were overexpressed in bacterial/fungal and viral keratitis (overlap up), as well as 216 genes which were specifically overexpressed in viral keratitis. Downregulated genes are visualized in the lower part of the heatmap. Each row represents one gene, each column represents one sample. The z-score represents a gene’s expression in relation to its mean expression by standard deviation units (red: upregulation, blue: downregulation). **(C)**: Functionally grouped network analysis of enriched Gene ontology biological processes, reactome, KEGG, and Wiki pathways in which the upregulated DEG were involved in. Enriched terms are visualized as nodes being linked based on the similarity of the genes linked to them. The node size represents the term enrichment significance (see legend). Representative terms of each group are labeled. The pie charts visualize the percentage of genes which were specific for bacterial/fungal (red), which were overexpressed in both bacterial/fungal and viral keratitis (grey), as well as genes which were overexpressed in viral keratitis (orange). **(D)** The top ten DEG are visualized for subnetworks from **(C)**. The height of the bar corresponds to mean expression and the error bar represents standard deviation. Detailed data for each gene including adjusted p-values are in **Supplementary Table 1**.

To assess the impact of corneal infection on the human cornea, we analyzed the transcriptional profile of human corneal specimens that were obtained during keratoplasty at our institution. 23,600 expressed genes were detected using unbiased MACE RNA sequencing (**Supplementary Table 1**). Principal component analysis (PCA) revealed a substantial impact of corneal infection compared to normal controls and to keratoconus with distinct differences between bacterial/fungal and viral etiology (**Figure 1A**). Clinically, 60% of the viral cases demonstrated inactive chronic keratitis. Strikingly, these samples still clustered together with the active viral cases, indicating long-lasting transcriptional changes and that the etiology of the infection was considerably more relevant than its activity. Our analysis revealed hundreds of genes that were specific to the etiology of corneal infection. Among them, we identified 561 genes that were specifically overexpressed in bacterial/fungal keratitis, 216 in viral keratitis, and 453 genes that were overexpressed in both (**Figure 1B**). Similarly, there were 325 genes that were only downregulated in bacterial/fungal keratitis, 43 in viral keratitis, and 29 in both (**Supplementary Table 2**). We also performed receiver operating curve (ROC) analyses for all overexpressed viral and bacterial/fungal genes to determine the sensitivity, specificity, positive predictive value, negative predictive value, and accuracy for viral or bacterial/fungal keratitis, respectively (**Supplementary Table 3**). 60 of the 216 viral genes and 327 of the 561 bacterial/fungal genes demonstrated an area under the curve (AUC) of at least 0.9. *MATR3* (AUC = 0.95), *CXCL9* (AUC = 0.95), and *KCNA3* (AUC = 0.90) were among the best candidate markers for viral keratitis, and *S100A8* (AUC = 1), *CXCL8* (AUC = 1), and *CCL20* (AUC = 1) were among the best candidates for bacterial/fungal keratitis.

To gain new insights into the infection-induced modulations of pathways in the human cornea, a pathway network analysis was performed (**Figure 1C**). The analysis revealed several subnetworks of signaling pathways, some of them highly affected in bacterial/fungal and others in viral infection. Pathways that were particularly affected in bacterial/fungal infection included neutrophil activation and degranulation (e.g., *CCL20* and *CXCR1*), leukocyte migration and chemotaxis (e.g., *S100A8* and *CXCL8*), phagocytosis (e.g., *CXCL8* and *FCER1G*), complement activation (e.g., *SERPINE1* and *C1QA*), as well as several cytokine pathways, including TNF and IL17 signaling (e.g., *MMP9* and *CXCL5*) (**Figures 1C, D**). In contrast, several T cell-related pathways, including T cell co-stimulation, Th1 and Th2 differentiation, T cell selection, and T cell receptor signaling, were predominantly affected by viral infection. These results demonstrate substantial alterations of biological pathways during corneal infection and reveal that the infection’s etiology is associated with a specific and long-lasting gene expression profile and pathway network, which is in accordance to previous findings using microarray technology (Chidambaram et al., 2017). These findings considerably expand our understanding about the human host response to corneal infectious disease and identify new potential therapeutic targets to reduce inflammation-induced tissue damage.

### The human cornea demonstrates a specific host response to viral infection

**Figure 2:**
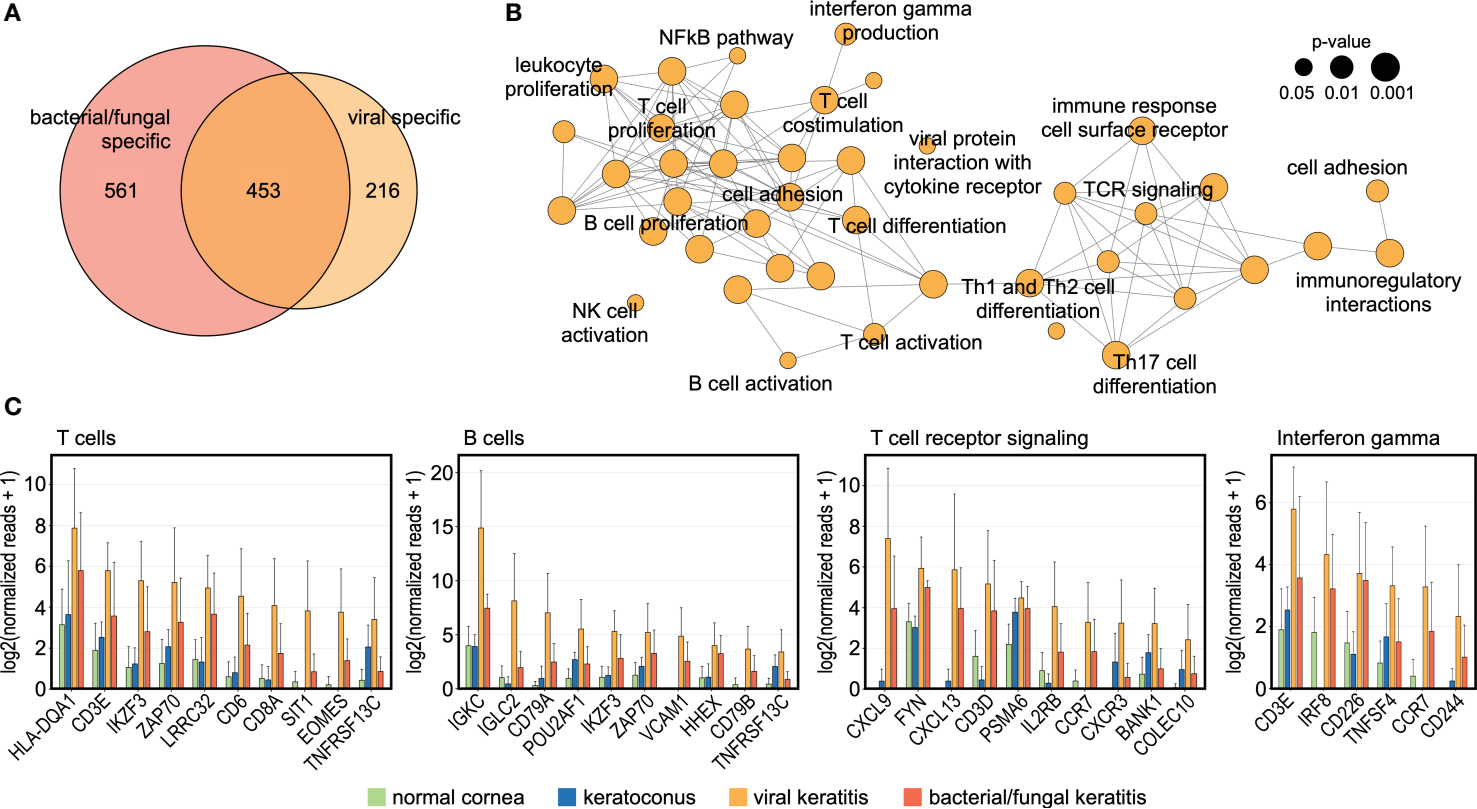
The human cornea demonstrates a specific host response to viral infection. **(A)**: Venn diagram demonstrating 216 genes which were only upregulated in viral keratitis but not in bacterial/fungal keratitis. **(B)**: Functionally grouped network analysis based on the 216 HSV-induced genes showing enriched pathways in the human cornea that are specific to viral keratitis. Each node represents one pathway and the nodes are connected based on the genes linked to them. The node size represents the term enrichment significance (see legend). Representative terms of each group are labeled. **(C)** The top ten DEG are visualized for subnetworks from **(B)**. The height of the bar corresponds to mean expression and the error bar represents standard deviation. Detailed data for each gene including adjusted p-values are in **Supplementary Table 1**.

To better understand the host response to viral corneal infections, we further focused our analysis on the 216 virus-specific genes (**Figure 2A**). A functionally grouped network analysis identified subnetworks of signaling pathways involved in the response to viral infections in the human cornea (**Figures 2B, C**), among them T cell related pathways (e.g., *IKZF3*, *ZAP70*, *CD8A*, and *SIT1*), T cell receptor signaling (e.g., *CXCL9, FYN*, and *CXCL13*), B cell related pathways (e.g., *IGKC, IGLC2*, and *CD79A*), and interferon gamma signaling pathways (e.g., *IRF8*, *TNFSF4*, and *CCR7*). These results further support a virus-specific host response that is driven by adaptive immunity and associated molecular signaling pathways. These findings may lead to new potential therapeutic targets to reduce immune-mediated corneal tissue damage in viral keratitis.

### Infectious keratitis involves pathogen-specific modulations of the corneal cellular profile

**Figure 3:**
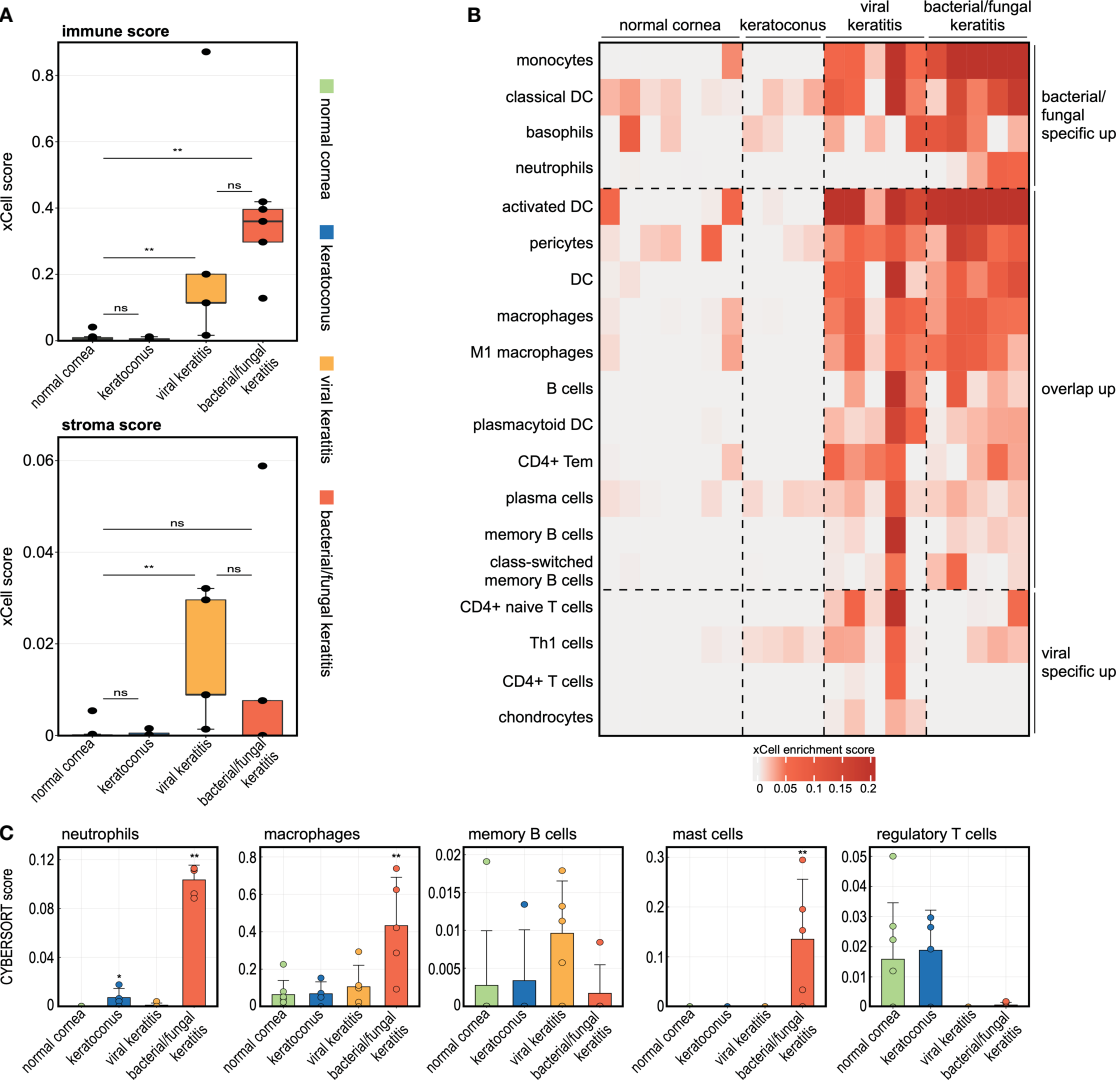
The corneal immune and stroma cell profile significantly changes in bacterial/fungal and viral keratitis. **(A, B)** The analyses are based on the tool xCell which uses gene expression profiles of 64 immune and stroma cell types to calculate relative cell type enrichment scores in a bulk transcriptome. **(A)**: The boxplots visualize summary scores for immune and stroma cell types. ** p < 0.01, ns: not significant. Each point represents one sample. **(B)** Heatmap illustrating xCell enrichment scores of cell types which were only significantly enriched in bacterial/fungal keratitis, which were enriched in both bacterial/fungal and viral keratitis, as well as cell types which were only enriched in viral keratitis. Each row represents one cell type, each column represents one sample. DC, dendritic cells; Tem, T effector memory cells; Th1, type 1 T-helper cells. **(C)** The tool CYBERSORTx was also applied to impute cell abundance from bulk RNA seq data. The height of the bar corresponds to the mean enrichment score, the error bar reflects the standard deviation. Each point is one sample. Stars indicate significant changes compared to the control group: * p < 0.05, ** p < 0.01.

Having characterized the infection-induced modulations in the human cornea on the gene and pathway level, we next explored the impact of corneal infection on the cell types present. We applied the xCell bioinformatics tool which allows to calculate relative cell type enrichment scores in a bulk transcriptome based on known gene expression profiles of 64 immune and stroma cell types (Aran et al., 2017). xCell revealed substantial differences in the cellular profile between corneal infection and normal controls (**Figures 3A, B**). While bacterial/fungal keratitis demonstrated a stronger increase of the immune score (summary score of all immune cell types assessed by xCell), which is consistent with the acute nature of the infection, viral keratitis was particularly characterized by an increase of the stromal score (summary score of all stroma cell types assessed by xCell), which may reflect the more chronic nature and scarring seen in patients with viral keratitis (**Figure 3A**). Our analysis further revealed that monocytes, dendritic cells, basophils, and neutrophils were specifically involved in bacterial/fungal keratitis, whereas T cells and Th1 cells played a key role in viral keratitis (**Figure 3B**). We also applied CYBERSORTx as a second cell type deconvolution method (Newman et al., 2019). In accordance with the xCell results, we also found a strong increase of CYBERSORTx scores for neutrophils and macrophages in bacterial/fungal keratitis (**Figure 3C**). Scores for mast cells were significantly increased in bacterial/fungal keratitis using CYBERSORTx, while only slightly increased values were found with xCell which were not significant (p = 0.215). The enrichment of regulatory T cells was decreased in viral and bacterial/fungal keratitis, a finding that may correspond to disturbed immunosuppression during infectious keratitis.

**Figure 4:**
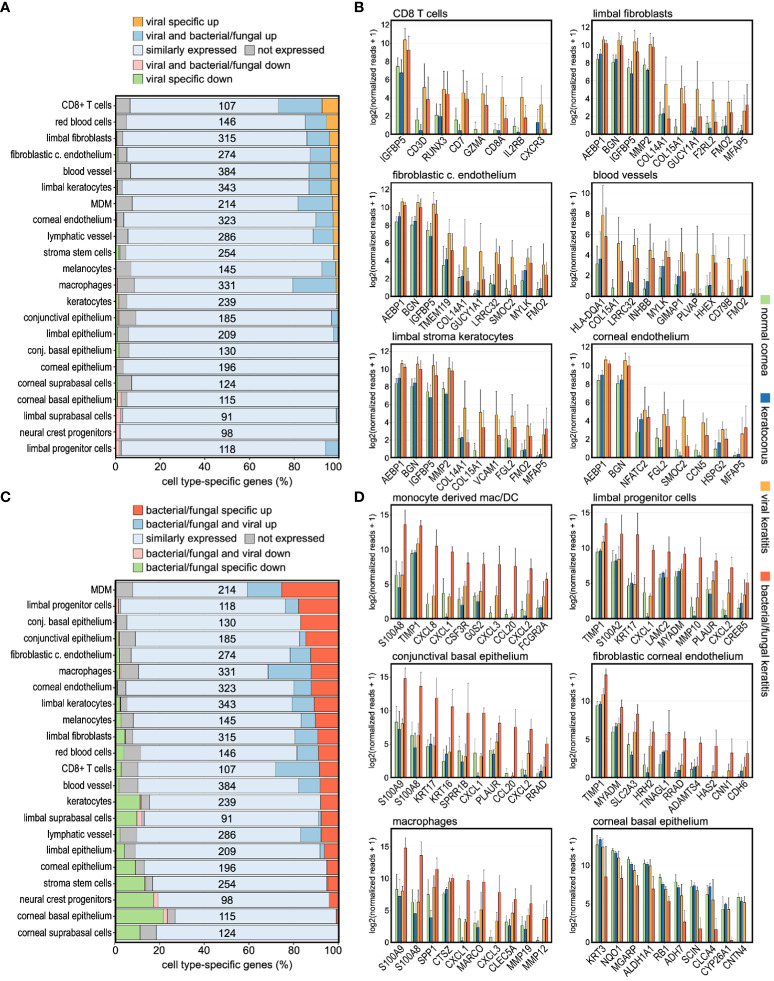
Infectious keratitis involves pathogen-specific modulations of the corneal cellular profile. **(A, C)**: Impact of **(A)** viral and **(C)** bacterial/fungal infection on the expression profile of corneal cell type-specific marker genes, as previously determined by single cell RNA sequencing. The relative number of marker genes per cell type is shown on the x-axis, whereas the absolute number of marker genes for each cell type is shown within the plot. Cell types are ordered based on the percentage of markers specifically upregulated in viral, or bacterial/fungal keratitis. **(B, D)**: The top ten differentially expressed cell type-specific marker genes are visualized for the most affected cell types in **(B)** viral and **(D)** bacterial/fungal keratitis. The height of the bar corresponds to mean expression and the error bar represents standard deviation. c., corneal; conj., conjunctival; DC, dendritic cells; MDM, monocyte-derived macrophages/DC. Detailed data for each gene including adjusted p-values are in **Supplementary Table 1**.

To further determine the cellular profile during corneal infection, we analyzed the expression of corneal cell type-specific marker genes, as previously determined by single cell RNA sequencing (Collin et al., 2021). Strikingly, the markers of CD8+ T cells, blood vessels, erythrocytes, corneal endothelial cells, limbal fibroblasts, and limbal keratocytes were most affected in viral keratitis, whereas in bacterial/fungal keratitis, the strongest alterations were seen in monocyte-derived macrophages/dendritic cells, macrophages, conjunctival epithelium, corneal endothelium, limbal progenitor cells, and limbal keratocytes (**Figures 4A, C**). The top ten differentially expressed genes of the highly regulated cell populations are visualized as bar graphs for each etiology (**Figures 4B, D**). We found, for example, that molecules involved in neutrophil recruitment such as *CCL20*, *CXCL1*, *CXCL2*, and *CXCL8* were mainly derived from corneal macrophages. This raises the possibility that tissue resident corneal macrophages may play a role in the initiation of neutrophil recruitment during bacterial/fungal keratitis, as previously described in other organs (Kolaczkowska and Kubes, 2013). These findings illustrate the general and pathogen-specific cellular modulations and possible interactions in the human cornea during bacterial/fungal and viral infection.

## Discussion

Infectious keratitis frequently leads to visual impairment and blindness. Usually, aggressive antimicrobial therapy can manage the acute infection, but the immune response often leads to host cell damage, resulting in scarring and opacification of the cornea and subsequent visual impairment. This indicates that modulation of the immune response may represent a promising therapeutic approach to improve the outcome of infectious keratitis.

Novel therapeutic strategies are urgently needed, but the molecular mechanisms of infectious keratitis are still poorly understood. Transcriptional profiling using RNA-seq allows us to examine disease mechanisms in an unbiased approach (Wolf et al., 2023). However, the technique has been mainly used with fresh tissue, which can be challenging to obtain in diseases like infectious keratitis. We recently demonstrated that a specialized 3’ RNA-seq method allows us to assess the transcriptional profile of archived FFPE corneal specimens that are available from histological biobanks (Boneva et al., 2020; Wolf et al., 2022b). Previous studies have applied microarray technology to investigate gene expression changes in infectious keratitis (Chidambaram et al., 2017). The more recent RNA-seq technology provides several advantages over microarray technology, including a more accurate and unbiased assessment of the gene expression profile (Liu et al., 2007). Microarray technology can be limited by cross-hybridization problems and is biased by the selection of probes, whereas RNA seq enables detection of all transcripts in a sample in an unbiased manner (Liu et al., 2007). Here, we applied RNA sequencing technology to assess the general and pathogen-specific molecular and cellular mechanisms during human corneal infection and identify 23,600 expressed genes corresponding to a 39% increase of detected genes compared to previous microarray analyses in infectious keratitis (Chidambaram et al., 2017).

We identified pathogen-specific transcriptional and cellular profiles that allow to distinguish bacterial/fungal from viral keratitis and provide new insights into the host response to infectious corneal disease. The virus-specific host response was driven by adaptive immunity and associated molecular signaling pathways, reflecting the chronic inflammatory nature of herpes simplex infection. In contrast, the bacterial/fungal-specific host response mainly involved innate immunity signaling pathways and cell types, consistent with the acute inflammation in these infections. Strikingly, the clinical activity of an infection appeared to have a much smaller impact on the corneal transcription profile than the infection’s etiology. These findings support the hypothesis that the corneal host response is tailored to different classes of pathogens. Elucidating these pathways could reveal new therapeutic targets to modulate inflammation and limit tissue damage in infectious keratitis.

Previously, the understanding of viral keratitis was mainly based on data derived from animal models (Bryant-Hudson et al., 2014; Koujah et al., 2019; Wang et al., 2020). Here we provide high-resolution molecular profiling of viral keratitis in humans that identifies the cellular and molecular driver of the human host response. Our results indicate an important role of T cells in this process. This finding is in accordance with studies in animal models of HSV keratitis, that reported T cells as the main coordinators in HSV keratitis (Bhela et al., 2017; Rajasagi and Rouse, 2019). Our results indicate that regulatory T cells decrease during infectious keratitis. Therapeutic agents which enhance the suppressive effect of regulatory T cells can effectively reduce the number of inflammatory cells, cytokines, and finally corneal tissue damage (Varanasi et al., 2017). We also found a strong upregulation of distinct chemokines and chemokine receptors in viral keratitis, such as *CXCL9* and *CXCR3*. CXCL9 is secreted by resident corneal cells and neutrophils. It accelerates the progression of viral keratitis (Molesworth-Kenyon et al., 2015; Tajfirouz et al., 2017) and mice lacking CXCL9 demonstrate reduced corneal infiltration by T cells (Wuest et al., 2006). Along these lines, mice that are deficient in CXCR3, the receptor for CXCL9, have less severe keratitis, indicating that the CXCL9 receptor is also a critical mediator of the pathological immune response in viral keratitis (Komatsu et al., 2008). Finally, matrix metalloproteinases (MMP) degrade the extracellular matrix and promote corneal neovascularization, a critical hallmark of viral keratitis. Animal studies have shown that MMP9 increases after HSV infection to promote corneal neovascularization (Lee et al., 2002). Depletion of neutrophils reduced MMP9 activation and pharmacological inhibition or genetic knockout of MMP9 reduced corneal neovascularization. Similarly, neutrophils may also mediate tissue damage in trachomatous scarring, potentially through the production of reactive oxygen species and matrix metalloproteinases (Barton et al., 2023). Some of the identified viral genes are also involved in other viral diseases, such as *IKZF3* which promotes proliferation of HIV-1-infected cells (Wei et al., 2023), *IRF8* which is involved in the induction of downstream antiviral genes in monocytes by DNA viruses (such as HSV-1) but not RNA viruses (Luo et al., 2022), and variants in *TNFSF4* which are associated with the risk of HCV infection (Fu et al., 2021). Thus, our results on human corneal tissue identify several mechanisms that are involved in the pathogenesis of viral infection and provide the foundation for a potential translation of previous findings from animal models to humans as novel therapeutic strategies for viral keratitis.

In bacterial keratitis, the therapeutic options remain limited despite a growing burden of disease, illustrating the urgent need for novel therapeutic approaches. However, this need can only be addressed with a detailed understanding of the complex biological mechanisms driving bacterial keratitis (Ung and Chodosh, 2021). Our results gained by RNA-seq in human samples of bacterial keratitis indicate an important role of the innate immune system, including neutrophils, monocytes, and macrophages. This finding is in accordance with previous studies that demonstrated that neutrophils are the most abundant cells in acute corneal inflammation, although monocytes and macrophages play critical supportive roles (Hazlett, 2004). We also observed that several neutrophil-attracting chemokines, including *CXCL3*, *CXCL5*, *CXCL6*, and *CXCL8*, were strongly upregulated in bacterial keratitis. Interestingly, these molecules are mainly derived from tissue resident corneal macrophages, which indicates that these cells may play a role in the initiation of neutrophil recruitment during bacterial/fungal keratitis, as previously described in other organs (Kolaczkowska and Kubes, 2013). Neutrophils release a variety of factors, including inflammatory cytokines, lysosomal enzymes, reactive oxygen species, and neutrophil extracellular traps, that target the bacterial invaders. Although these mechanisms are critical for the acute clearance of pathogens, they are also highly toxic to corneal host cells, including the corneal stroma, in which they digest structural molecules like collagens (Hazlett, 2004). While the initial innate immune response is crucial, its persistent dysregulation is at least partly responsible for subsequent corneal tissue damage and efforts to modulate this persistent dysregulation may be beneficial for patients with bacterial keratitis. Our results also demonstrated a strong upregulation of *CCL20*. In a spinal cord injury animal model, a CCL20 neutralizing antibody reduced leukocyte infiltration and improved functional outcome (Hu et al., 2016). These results indicate that CCL20 may represent a target candidate to modulate persistent dysregulation of the innate immune response in bacterial keratitis. Our results further revealed a strong upregulation of *S100A8/A9* (calprotectin), an antimicrobial and pro-inflammatory protein complex serving as a biomarker for several inflammatory diseases (Schlecht et al., 2020). In a mouse model of intra-abdominal candidiasis, treatment with an S100A9 inhibitor stopped collateral inflammatory tissue damage and S100A9-deficient mice demonstrated increased survival compared to wild-type littermates (Shankar et al., 2021). These findings strongly suggest that S100A8/9 inhibition may positively modulate the balance between pro- and anti-inflammatory control in bacterial keratitis to reduce collateral damage of corneal tissue. In addition, we found an increased expression of *MMP1* and *MMP9* in bacterial keratitis. It has been shown that cytokine-induced MMP9 activation in human corneal epithelial cells may contribute to corneal matrix degradation and that doxycycline can suppress MMP9 activation (Li et al., 2001). Finally, our analysis identified the IL17 pathway as one of the key mediators of the host response to bacterial and viral infections. IL17 induces the expression of antimicrobial peptides, cytokines, and chemokines that help recruit and activate immune cells (McGeachy et al., 2019). The IL17 cytokine family promotes the acute clearance of pathogens but also drives inflammatory pathology and is involved in a variety of inflammatory diseases, including cardiovascular and neurological diseases (Mills, 2023). This led to the development of therapeutic antibodies which demonstrated efficacy in several diseases (Mills, 2023). In bacterial and fungal keratitis, a role of IL17 signaling has previously been reported in corresponding animal models (Qin et al., 2019; Me et al., 2020). Mice deficient in IL17 signaling exhibited impaired neutrophil recruitment and clearance of bacteria or fungi from the cornea (Taylor et al., 2016; Me et al., 2020). However, uncontrolled IL17 responses led to corneal damage, highlighting the need for regulation. Polymorphisms in IL17 and related genes are linked to differences in susceptibility and severity of keratitis, suggesting a genetic basis for IL17 response variability (Konda et al., 2022). These findings indicate a possible therapeutic potential of IL17 pathway modulation in infectious keratitis. Neutralizing IL17 could limit persistent inflammation, whereas boosting IL17 immunity may enhance infection control when needed. Our results identify several potential therapeutic targets for human bacterial keratitis that could critically expand the currently limited therapeutic options for this growing disease burden. Clinical translation will require to determine optimal treatment timing with the goal to beneficially strike the balance between a pro-inflammatory response to eliminate the pathogen and anti-inflammatory control to prevent damage to corneal host cells.

The host immune response often leads to collateral cell damage that results in corneal scarring and significant visual impairment. Corneal scarring can also be observed in keratoconus, a non-infectious structural disease of the cornea. To address the impact of the corneal immune response on tissue damage, we compared the transcriptional profiles of infectious keratitis to keratoconus samples, revealing massive differences between these entities on the gene, pathway, and inferred cell level, further supporting the specificity of our findings for viral and bacterial/fungal keratitis. Several factors are involved in corneal scarring, including the severity and duration of the infection as well as the pathogen type. For example, herpes simplex virus is intrinsically more prone to induce scarring. Damage to the limbal stem cells, which normally replenish and maintain the corneal epithelium, leads to scarring by impairing normal epithelial regeneration and promoting conjunctivalization of the cornea. Corneal neovascularization supplies inflammatory cells, pro-fibrotic factors, and myofibroblast precursors that promote scarring (Mohan et al., 2022). Genes which influence the inflammatory and wound healing responses are linked to differences in scarring susceptibility between individuals (Yeung et al., 2022). It has been shown that an uncontrolled or chronic inflammation leads to tissue damage and release of pro-fibrotic mediators like TGF-beta (Torricelli et al., 2013; Yang et al., 2016). Indeed, we found a strong upregulation of *TGFB1* in both bacterial/fungal and viral human keratitis, indicating a potential application of TGF-beta inhibitors to reduce corneal scaring. Another important process contributing to corneal scarring is abnormal corneal wound healing, which involves myofibroblast activation and excess extracellular matrix deposition (Yeung et al., 2022) which are mediated by growth factors like PDGF and CTGF (Sriram et al., 2013). In our study, we found a significant upregulation of *CTGF* in bacterial/fungal keratitis but not in viral keratitis, suggesting a potential therapeutic application of CTGF modulators in bacterial/fungal keratitis. More samples including specimens infected by various pathogens are needed to validate these findings and to further refine the line-up of potential diagnostic biomarkers and therapeutic targets.

By integrating the transcriptional profiles of bacterial/fungal and viral keratitis, we were able to identify 216 genes that were specifically overexpressed in viral keratitis and 561 genes that were specific to bacterial/fungal keratitis. We identified 60 viral genes and 327 bacterial/fungal genes with an AUC of at least 0.9, among them *MATR3*, *CXCL9*, and *KCNA3* for viral keratitis, and *S100A8*, *CXCL8*, and *CCL20* for bacterial/fungal keratitis. These genes represent potential biomarkers that may help in diagnostic evaluation of infectious keratitis, a fundamental and time-critical process to initiate appropriate therapy without unnecessary delay. Future studies with larger sample sizes may help to differentiate bacterial and fungal keratitis. In addition, the markers may also serve to monitor the treatment response. A PCR- or ELISA-based biomarker panel that captures only a few selected biomarkers from small sample amounts like corneal scraping or tear fluid could provide results at the point of care within minutes during clinical routine. However, further studies are needed to achieve this goal. Our work provides the proof-of-concept that in principle a differentiation between bacterial/fungal and viral keratitis is possible using high-resolution molecular profiling.

In summary, this study provides new highly resolved insights into the human corneal host response to viral and bacterial/fungal infection, including etiology-specific gene expression profiles, signaling pathways, and cell types that were mainly involved in innate immunity in bacterial/fungal keratitis and adaptive immunity in viral keratitis. We identified several specific genes and pathways involved in the host response to infectious keratitis, including CXCL9, CXCR3, and MMP9 for viral, and S100A8/A9, MMP9, and the IL17 pathway for bacterial/fungal keratitis. The genes, pathways, and cell types characterized could reveal innovative strategies to diagnose or monitor disease progression, and develop immunomodulatory treatments for keratitis. Etiology-specific genes may be valuable as diagnostic biomarkers for faster diagnosis and initiation of appropriate treatment regimens. Deciphering the complex interplay between pathogens and host in the cornea will be critical to overcome blindness from infection, improving outcomes, and quality of life for patients. Overall, these results highlight the promise of transcriptomics to advance precision medicine approaches in infectious keratitis.

## Data availability statement

The datasets presented in this study can be found in online repositories. The names of the repository/repositories and accession number(s) can be found below: https://www.ncbi.nlm.nih.gov/geo/, GSE241715.

## Ethics statement

Written informed consent was obtained from all participating patients. Ethical approval was granted from the Research Ethics Committee of the University of Freiburg, Germany (reference number: 434/20). The studies were conducted in accordance with the local legislation and institutional requirements. The participants provided their written informed consent to participate in this study.

## Author contributions

TL: Conceptualization, Data curation, Funding acquisition, Investigation, Methodology, Project administration, Resources, Supervision, Validation, Visualization, Writing – original draft, Writing – review & editing. PK: Conceptualization, Data curation, Formal analysis, Funding acquisition, Investigation, Methodology, Writing – review & editing. GS: Conceptualization, Methodology, Project administration, Supervision, Writing – review & editing. CA-H: Conceptualization, Methodology, Validation, Writing – review & editing. PM: Conceptualization, Supervision, Validation, Writing – review & editing. CL: Conceptualization, Methodology, Validation, Writing – review & editing. TR: Resources, Supervision, Writing – review & editing. JW: Conceptualization, Data curation, Formal analysis, Investigation, Methodology, Software, Validation, Visualization, Writing – original draft, Writing – review & editing.

## Supplementary material

Supplementary Figure 1 Examples of clinical **(A, E, I, M)** and histological images of patients enrolled into the trial. Clinical diagnoses were confirmed using histology. **(A–D)** Healthy corneas obtained from enucleated globes (see **Table 1** for details) served as controls. **(E–H)** Corneas from patients with keratoconus were used as a second control group. **(I–L)** Clinical images and corresponding histological images of a herpetic scar are shown. Vessels in the cornea can also be identified histologically, here surrounded by numerous plasma cells (inset in **(L)**). **(M–P)** Clinical images and corresponding histological images of an acute bacterial keratitis are shown. A distinct massive accumulation of polymorphonuclear neutrophils (PMNs) (P) is seen in acute bacterial keratitis **(M)**.
