# Supplementary material for: Transcriptional profiling specifies the pathogen-specific human host response to infectious keratitis
Source: Front Cell Infect Microbiol. 2024 Jan 11;13:1285676. doi: 10.3389/fcimb.2023.1285676 (PMC10808294; doi:10.3389/fcimb.2023.1285676)
Supplement: Supplementary Figure 1 — Examples of clinical (A, E, I, M) and histological images of patients enrolled into the trial. Clinical diagnoses were confirmed using histology. (A–D) Healthy corneas obtained from enucleated globes (see Table 1 for details) served as controls. (E–H) Corneas from patients with keratoconus were used as a second control group. (I–L) Clinical images and corresponding histological images of a herpetic scar are shown. Vessels in the cornea can also be identified histologically, here surrounded by numerous plasma cells (inset in (L)). (M–P) Clinical images and corresponding histological images of an acute bacterial keratitis are shown. A distinct massive accumulation of polymorphonuclear neutrophils (PMNs) (P) is seen in acute bacterial keratitis (M). [file Image_1.pdf]

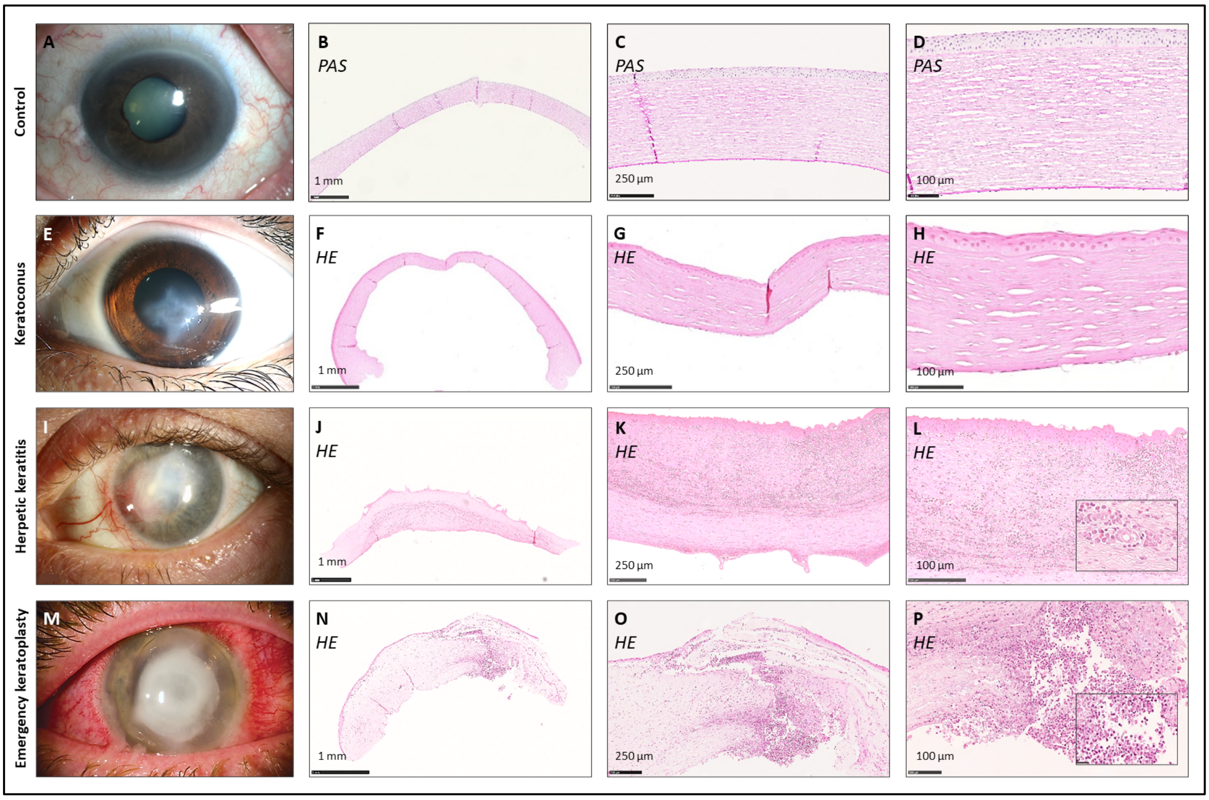

Supplementary Figure 1: Examples of clinical (A, E, I, M) and histological images of patients enrolled into the trial. Clinical diagnoses were confirmed using histology. (**A-D**) Healthy corneas obtained from enucleated globes (see Table 1 for details) served as controls. (**E-H**) Corneas from patients with keratoconus were used as a second control group. (**I-L**) Clinical images and corresponding histological images of a herpetic scar are shown. Vessels in the cornea can also be identified histologically, here surrounded by numerous plasma cells (inset in (**L**)). (**M-P**) Clinical images and corresponding histological images of an acute bacterial keratitis are shown. A distinct massive accumulation of polymorphonuclear neutrophils (PMNs) (**P**) is seen in acute bacterial keratitis (**M**).
